# Association of Mannose-binding Lectin Polymorphisms with Tuberculosis Susceptibility among Chinese

**DOI:** 10.1038/srep36488

**Published:** 2016-11-04

**Authors:** Cheng Liu, Tao He, Yanxiao Rong, Fengjiao Du, Dongxing Ma, Yujie Wei, Zhiqin Mei, Yuling Wang, Haibin Wang, Yuehua Zhu, Zongde Zhang, Li Zheng, Xueqiong Wu, Huiliang Liu, Wenjun Ding

**Affiliations:** 1 Medical Experimental Center, General Hospital of Chinese People’s Armed Police Forces, Beijing, P. R. China; 2 Department of Orthopedics (307 Hospital of PLA), Affiliated Hospital of Academy of Military Medical Sciences, Beijing, P. R. China; 3 Beijing Institute of Biotechnology, Beijing, P. R. China; 4 Laboratory of Environment and Health, College of Life Sciences, University of Chinese Academy of Sciences, Beijing, P. R. China; 5 Department of Cardiology, General Hospital of Chinese People’s Armed Police Forces, Beijing, P. R. China; 6 Department of Tuberculosis, The Fifth Hospital of Shijiazhuang, Shijiazhuang City, P. R. China; 7 Beijing Key Laboratory for Drug Resistance Tuberculosis Research, Beijing Chest Hospital, Capital Medical University, Beijing Tuberculosis and Thoracic Tumor Research Institute, Beijing, P. R. China; 8 Army Tuberculosis Prevention and Control Key Laboratory, Institute for Tuberculosis Research, the 309th Hospital of Chinese PLA, Beijing, P. R. China

## Abstract

Tuberculosis (TB) is caused by infection of *Mycobacterium tuberculosis*. Host genetic variability is an important determinant of the risk of developing TB in humans. Although the association between *MBL2* polymorphisms and TB has been studied in various populations, the results are controversial. In this study four functional single-nucleotide polymorphisms (SNPs, H/L, X/Y, P/Q and A/B) across the *MBL2* gene were genotyped by direct DNA sequencing of PCR products in a case-control population of Chinese Han origin, consisting of 1,020 patients with pulmonary TB and 1,020 controls. We found that individuals carrying variant allele at A/B (namely BB or AB genotypes) was associated with increased susceptibility to TB (odds ratios [OR] = 1.57, 95% confidence interval [CI] 1.30–1.91, *P* = 1.3 × 10^−6^). Additionally, LYPB haplotype showed a significant association with increased risk of TB (OR = 1.54, 95% CI 1.27–1.87, *P* = 4.2 × 10^−6^; global haplotype association *P* = 3.5 × 10^−5^). Furthermore, individuals bearing low- or medium- MBL expression haplotype pairs had an increased risk of TB (OR = 1.56, 95% CI 1.29–1.90, *P* = 1.4 × 10^−6^). Thus, the reduced expression of functional MBL secondary to having *MBL2* variants may partially mediate the increased susceptibility to TB risk.

Tuberculosis (TB) is caused by the infection of *Mycobacterium tuberculosis* (*Mtb*). *Mtb* presumably infects a third of the world’s population, with China has the second-largest number of infected cases^1^. Despite the improved treatment during the past decades, TB remains the first leading cause of global death from infectious diseases. The host-pathogen interactions and environmental factors may contribute to TB. Furthermore, Host genetic variability is an important determinant of the risk of developing TB in humans^2^. Unraveling the genetic factor contributing to the pathogenesis of TB may lead to improved treatment and prevention of this disease^3^.

The innate immune response in human activates the T helper 1 (Th1)-type immune system and plays an important role in the host’s defense against the development of TB^4^. Many studies have reported the association between TB and genetic polymorphisms related to human innate immunity^5^. Mannose-binding lectin (MBL) is a member of the collectin family that recognizes pathogens by its carbohydrate-recognition domains^6^. MBL is an acute-phase serum protein, which is synthesized in the liver and circulates as oligomers complexed with MBL-associated serine proteases (MASPs)^7^. MASPs binds to the sugar moieties on the surface of a pathogen, and then are activated to initiate the lectin pathway of complement activation, resulting in opsonization and phagocytosis or lysis of pathogens. In addition, MBL can regulate inflammatory responses and immune activation^7^.

MBL is encoded by the *MBL2* gene, which is located on chromosome 10. Six single-nucleotide polymorphisms (SNPs) in exon 1 (codon 52 *A*/*D*, codon 54 *A*/*B*, and codon 57 *A*/*C*) and in the promoter and 5′-untranslated regions (nt −550 *H*/*L*, nt −221 *X*/*Y*, and nt + 4 *P*/*Q*) of the *MBL2* gene are associated with serum levels and/or functions of MBL. These genetic variations are associated with a wide variety of diseases, including respiratory tract infections^3^,^8^,^9^,^10^,^11^. However, in previously reported studies *MBL2* polymorphisms have conflicting results showing protection from or susceptibility to TB^5^. For example, low levels of MBL (associated with variant alleles at the promoter and exon 1 regions of the *MBL2* gene) were reported to protect against tuberculosis^12^,^13^, supporting the hypothesis that MBL binding can enhance the uptake of intracellular pathogens by phagocytes, and then promote infection^12^,^14^. Other investigators instead claim that protection against the disease is associated with high levels of MBL (controlled by the wild *MBL2* alleles)^15^,^16^,^17^,^18^. In the Chinese Han population, no convincing evidence of association between *MBL2* sequence variants and TB was observed^19^,^20^,^21^,^22^,^23^. For example, in a meta-analysis totally including 880 TB patients and 959 controls of Chinese origin, Shi J and colleagues reported that variants at A/B were associated with increased susceptibility to TB^20^. Additionally, Chen M and colleagues found that variants in Y/X was associated with increased susceptibility to TB among Chinese^21^,^22^. However, most recently Wu L reported that variants in H/L, but not Y/X, P/Q or A/B, were associated with decreased susceptibility to TB among Chinese^23^. Conflicting results are not unexpected in association studies for several reasons, including small sample size, marginal statistical significance, detection of genotypes, or ethnic heterogeneity.

Therefore, here we conducted a genetic association study in a large case-control population of Chinese Han origin (totally consisting of 1,020 patients with TB and 1,020 controls), to better define the association between the *MBL2* SNPs and TB.

## Results

### Population Characteristics

The selected characteristics of patients with TB and control subjects in this study are shown in Table 1.

The study consists of 1,020 patients with TB and 1,020 control subjects. The male/female ratio of patients with TB was 1.10, and the mean age was 43.2 years (±SD, 14.9) (Table 1). For the 1,020 controls, the male/female ratio was 1.02, and the mean age was 44.3 years (±SD, 15.0). Controls were comparable with cases with regard to mean age and gender (all *P* values > 0.05).

The genotyping results are presented in Table 2. The observed genotype frequencies for the four polymorphisms conformed to the Hardy-Weinberg equilibrium in controls (all *P* values > 0.05). Consistent with the previous reports^11^,^24^, the codon 52 (A/D) and 57 (A/C) variants were not observed in our ethnic northern Chinese individuals. The frequencies of variant alleles L, X, Q and B in the controls were 48.7%, 14.5%, 13.2% and 14.0%, respectively, similar to those reported in other southern or northern Chinese subpopulations^11^,^24^.

### Polymorphisms and Risk of TB in this study

On the basis of unconditional logistic regression analysis with adjustment for known confounding factors including age and gender, a statistically significant association with the susceptibility to TB was observed with the condon 54 variant. Subjects homozygous and heterozygous for variant B allele (i.e. B/B plus A/B genotype) had an increased susceptibility to TB compared to those homozygous for the wild-type A allele (OR = 1.57, 95% CI 1.30–1.91, *P* = 1.3 × 10^−6^; simulation number = 10,000,000; Table 2). The association was still significant even after correction for multiple comparisons. None of the remaining three variants (H/L, Y/X and P/Q) were found to be significantly associated with susceptibility to TB. Logistic regression models including random effects obtained results similar to those obtained in models including fixed-effects (Table 2).

In addition to single SNP analysis, haplotype analysis was also performed for the *MBL2* gene. We identified 5 haplotypes (HYPA, LYPB, LXPA, LYQA and LYPA) using the PHASE program, with the frequencies of these haplotypes greater than 5% in both cases and controls. The frequencies of these 5 haplotypes in controls ranged from 51.3% (HYPA haplotype) to 7.0% (LYPA haplotype), similar to those reported in other Asian populations of Japanese and Vietnamese^16^.

In haplotype analysis, only LYPB showed a significant association with increased risk of TB (OR = 1.54, 95% CI 1.27–1.87; *P* = 4.2 × 10^−6^; global haplotype association *P* = 3.5 × 10^−5^; simulation number = 10,000,000; Table 3). Furthermore, we grouped cases and controls by harboring haplotypes associated with high, medium, or low MBL expression^25^, to evaluate whether haplotype pairs associated with various levels of MBL expression were associated with susceptibility to TB risk. In 1,020 patients with TB, 618 (60.6%) were in the high expression group, 298 (29.2%) in the medium group, and 104 (10.2%) in the low group. In 1,020 controls, this distribution was 723 (70.9%), 234 (22.9%) and 63 (6.2%), respectively (OR = 1.56, 95% CI 1.29–1.90, *P* = 1.4 × 10^−6^, with adjustment for age and gender; simulation number = 10,000,000; Table 2), implicating that the individuals bearing medium or low expression haplotype pairs had an increased risk of TB and that the MBL deficiency might play a potential role in susceptibility to TB.

The associations between the four *MBL2* polymorphisms and susceptibility to TB were further examined with stratification by age and gender, with no differences observed for *MBL2* polymorphisms individually or in haplotype between stratums (all *P* values > 0.05, test for homogeneity).

## Discussion

Recent studies on the genetic association between SNPs in the *MBL2* gene and patients with TB of Chinese origin have generated different and even contradictory results. These studies have small sample size, usually involving about 200 or less PTB patients of Chinese origin^19^,^20^,^21^,^22^,^23^, which might lead to artificial associations. In addition, the four functional SNPs in the *MBL2* gene is in linkage disequilibrium (LD) and more appropriate to be investigated together (in both haplotype and haplotype pairs). Moreover, marginal statistical significance reported in these association studies means that poor reproducibility of their results is not unexpected, given the concern over the possible unreliability of case-control studies^26^.

In this study, we investigated all of the four functional SNPs in the *MBL2* gene, individually or in haplotype or haplotype pairs, in a large case-control population of Chinese Han origin, totally consisting of 1,020 patients with TB and 1,020 controls. This is the first study to investigate the association between *MBL2* polymorphisms with pulmonary TB susceptibility in northern Han Chinese. By genotyping all of the four functional SNPs in the *MBL2* gene in the case-control population of relatively large sample size, we found that one SNP (A/B) was associated with susceptibility to TB (*P* = 1.3 × 10^−6^). Furthermore, we found that the LYPB haplotype showed a significant association with increased risk of TB in northern Han Chinese (*P* = 4.2 × 10^−6^). In addition, individuals bearing haplotype pairs indicating medium or low *MBL2* expression had an increased risk of TB (*P* = 1.4 × 10^−6^). We noted that Wu *et al*. have reported that LYPB haplotype was associated with TB in southern Chinese^23^. However, frequencies of *MBL2* haplotypes in Wu’s study were remarkably different from those in Chinese subjects observed in other studies. For example, frequencies of HYPA, LYPB, LXPA, LYQA and LYPA were 51.3%, 14.0%, 14.5%, 13.2% and 7.0% respectively in the controls in the present study, similar to the frequency distribution of these haplotypes in southern and northern Chinese in the 1000 genomes project. However, in the controls in Wu’s study frequencies of HYPA, LYPB, LXPA, LYQA and LYPA were 29.3%, 0.9%, 7.1%, 3.6% and 37.1% respectively. Based on the large sample size, haplotype pairs analyses and small *P* values, our results may be closer to the real situation of *MBL2* polymorphisms in TB susceptibility.

The *MBL2* gene encodes a homotrimeric molecule harboring a carbohydrate recognition domain and a collagenous tail^27^. The variant B allele can impair the formation of a triple helix in the collagenous tail, and therefore disrupts MBL polymerization and leads to enzymatic degradation and functional deficiency of this protein^28^. The other SNPs, H/L, X/Y, and P/Q, also play roles in determining functional MBL concentration, among which the X allele shows the lowest transcriptional activity^25^,^29^,^30^. When in haplotype, these SNPs form three defective haplotypes (LYPB, LYQC and HYPD, with the latter two absent among Chinese), and five functional haplotypes with different expression levels (a low-producing LXPA haplotype, an intermediate-producing LYPA haplotype, and two high-producing haplotypes LYQA and HYPA)^30^. The results that B allele, LYPB haplotype, and medium- or low-expression haplotype pairs were associated with increased risk of developing TB (Tables 1, 2 and 3) suggest that MBL deficiency possibly plays a role in susceptibility to TB.

There is biological plausibility for our observed genetic associations, although the exact mechanism by which MBL deficiency is associated with increased susceptibility to TB requires further investigation. Previous studies have revealed that MBL recognizes *M. tuberculosis* by direct interaction, resulting in lectin pathway activation, agglutination of bacteria and enhancement of phagocytosis^31^,^32^. Additionally, the serum samples from carriers of haplotype pairs associated with high MBL levels (HYA/HYA) display significantly higher antibacterial activity of neutralization than did those associated with lower MBL levels, and the activity is mediated by MBL^15^. Therefore, one speculative interpretation of our results is that low serum concentrations of functional MBL caused by haplotype pairs indicating medium or low *MBL2* expression might result in reduced neutralization and favor the survival of *M. tuberculosis*.

There are several potential limitations in the present study. The first, the patients with TB were selected from the hospital and the controls were recruited from the community population, which means that inherent selection bias cannot be completely excluded. However, by matching on age and gender and by analyzing data with further stratification, potential confounding effect might have been minimized. The second, some of the control subjects used in this study may be asymptomatic with latent TB infection. Therefore, we cannot directly specify which stage of TB, infection of *Mtb* or development of active disease, was more affected by *MBL2*. The third, although the highly significant association between MBL deficiency and increased susceptibility to TB derives from a biologically based *a priori* hypothesis, the initial findings presented here should be independently verified in other subpopulations of ethnic Chinese origin (e.g. southern Chinese) or of different ancestry.

In summary, our results reveal an association between genetic MBL deficiency and increased susceptibility to TB among northern Chinese, and provide supports for the importance of MBL in the pathogenesis of TB. Knowledge of genetic factors contributing to the pathogenesis of TB revealed in this study could lead to improved treatment and prevention of this disorder.

## Methods

### Ethics statement

Written informed consent was obtained from all participants involved in this case-control genetic association study, and the study was approved by the Medical Ethics Committee of the PLA 307 Hospital (Beijing, China) and the Fifth Hospital of Shijiazhuang (Shijiazhuang City, Hebei province, China). All the experiments were performed in accordance with the relevant guidelines and regulations.

### Subjects and Samples

A total of 1,020 patients with pulmonary TB were recruited at the Fifth Hospital of Shijiazhuang (Shijiazhuang City, Hebei province, China), between January 2010 and January 2016. All patients with pulmonary TB were newly diagnosed based on smear or culture positive for *M. tuberculosis* and chest radiography. Patients with confirmed diagnosis of extrapulmonary TB were excluded. The response rate for case patients was 93%. A total of 1,020 healthy adults frequency matched to the TB patients on the basis of age and gender were recruited as control subjects during the same time period as the TB patients were collected. The response rate for controls was 91%. All participants were unrelated northern Han Chinese, and had no clinical histories of diabetes mellitus, HIV infection, or receipt of immunosuppressive therapy. The authors had access to identifying information during and after data collection.

### Extraction of Genomic DNA

About 1-mL volume of peripheral blood samples was taken from each participant, and were frozen at −20 °C. Total genomic DNA was extracted from the samples using the Whole Blood DNA Extraction Kit (Tiangen Biotech, Co., Ltd, Beijing, China), according to the manufacturer’s instructions. DNA was dissolved in 0.1 × TE buffer (10 mM Tris-1 mM EDTA, pH 8.0) and stored at −20 °C.

### SNP Genotyping

The promoter polymorphisms at nt −550 (H/L, rs11003125), −221 (Y/X, rs7096206), and +4 (P/Q, rs7095891) and the structural polymorphism at nt +230 (codon 54 A/B, rs1800450) of the *MBL2* gene were selected for genotyping by use of PCR direct sequencing in the case-control population. The PCR primers were designed using the web-based software Primer3 (available at: http://primer3.ut.ee/). The forward primer 5′-ATGGGAGGAGGATTCAAGG-3′ and reverse primer 5′-CCTTGTGACACTGCGTGACT-3′ were used for amplifying the target region containing the *MBL2* H/L and Y/X variants. The forward primer 5′-CGGTCCCATTTGTTCTCACT-3′ and reverse primer 5′-CACAAACTGCTGTGTGGAAT-3′ were used for amplifying the target region containing the *MBL2* P/Q and A/B variants. Then the PCR products were sequenced by an ABI 3730 sequencer. A nucleotide was identified as a candidate polymorphism by an observer using the PolyPhred program (available at: http://droog.gs.washington.edu/polyphred/) and confirmed by another observer independently. Genotyping of all samples was performed in a blinded manner so that the case or control status was unknown. The call rates were 100% for all SNPs. The accuracy of genotyping data for each SNP was validated by masking, choosing at random, and resequencing 5% of the samples from the opposite strand.

### Statistical Analyses

Comparisons of gender and age between TB patients and controls were performed using the one-sided *χ*^*2*^ test (SPSS software, version 10.0). Differences of mean age between TB patients and controls were analyzed using the unpaired *t* test (SPSS software, version 10.0). Genotype frequencies for each SNPs were determined by gene counting. The significance of deviations from the Hardy-Weinberg equilibrium was tested using the online software SNPStats (http://bioinfo.iconcologia.net/SNPstats_web). Unconditional logistic regressions under a dominant inheritance model were used due to the limited sample size, to calculate *P* values, Odds ratios (ORs), and 95% confidence intervals (CIs) for assessing the association between SNPs and disease risk, adjusting for age and gender (fixed effects). In additional analyses, age and gender were treated as random effects in the logistic regression to deal with the uncertainties with more flexibility. The glm-function and glmer-function in the lme4 package (version 1.1–12)^33^ of R (version 3.3.1)^34^ were used to fit the models with fixed effects and random effects, respectively. To show the robustness of the conclusions, we performed resampling statistics as previously reported^35^. The *P* values, ORs, and 95% CIs in single SNP analyses were calculated by a bootstrapping test with the number of simulations was set to 10,000,000. As to multiple comparisons, the correction factor n(m-1) (in which there are n loci with m alleles) was used to correct the significance level.

The haplotypes of the *MBL2* gene of the case-control population were assigned by the PHASE program^36^. *P* values of association between haplotypes and disease risk were evaluated using the haplo.score-function^37^ in the haplo.stats package (version 1.7.7) of R, with the number of simulations was set to 10,000,000. ORs and 95% CIs were calculated for each haplotypes using the haplo.glm-function^38^ in the haplo.stats package. All *P* values and ORs (95% CI) were calculated by adjusting for age and gender. Analyses of haplotype pairs were similar to those of single SNPs.

Potential modification of the effect of the SNPs on TB risk was assessed for age and gender by the addition of interaction terms in the logistic model and by separate analyses of subgroups of subjects determined by these factors. A homogeneity test was used to detect the difference of ORs within each subgroups.

All tests are two-tailed unless otherwise indicated. *P* values less than 0.05 were considered statistically significant.

## Additional Information

**How to cite this article**: Liu, C. *et al*. Association of Mannose-binding Lectin Polymorphisms with Tuberculosis Susceptibility among Chinese. *Sci. Rep*. **6**, 36488; doi: 10.1038/srep36488 (2016).

**Publisher’s note:** Springer Nature remains neutral with regard to jurisdictional claims in published maps and institutional affiliations.

## Figures and Tables

**Table 1:** The clinical characteristics of patients with tuberculosis and control subjects included in this study.

Characteristics	Cases (n = 1,020)	Controls (n = 1,020)	*P*
Age, mean in years ±SD	43.2 ± 14.9	44.3 ± 15.0	0.13
<44, n (%)	519 (50.9)	494 (47.6)	0.27
≥44, n (%)	501 (49.1)	526 (52.4)	
Gender			
Female, n (%)	486 (48.4)	504 (49.4)	0.43
Male, n (%)	534 (51.6)	516 (50.6)	

Cases are patients with tuberculosis.

Comparisons of gender and age distributions between cases and controls were performed by use of the one-sided *χ*^*2*^ test. Differences of mean age between cases and controls were analyzed by use of an unpaired *t* test.

**Table 2:** The distribution of mannose-binding lectin (MBL) genotypes and haplotype pairs associated with various levels of MBL expression in patients with tuberculosis and control subjects.

Polymorphisms	No. of cases^a^ (n = 1,020)	No. of controls^a^ (n = 1,020)	OR (95% CI)^b^	*P*^b^	OR (95% CI)^c^	*P*^c^
H/L	276/498/246	231/532/257	1.06 (0.86–1.29)	0.60	1.07 (0.90–1.27)	0.53
Y/X	27/214/779	25/246/749	0.86 (0.71–1.06)	0.14	0.85 (0.72–1.01)	0.13
P/Q	12/210/798	11/248/761	0.83 (0.67–1.01)	0.065	0.82 (0.69–0.98)	0.062
A/B	30/345/645	14/258/748	1.57 (1.30–1.91)	1.3 × 10^−6^	1.59 (1.36–1.87)	2.2 × 10^−6^
Expression groups	104/298/618	63/234/723	1.56 (1.29–1.90)	1.4 × 10^−6^	1.59 (1.35–1.86)	1.8 × 10^−6^

OR, odds ratio; CI, confidence interval.

H/L, Y/X, and P/Q are functional promoter polymorphisms at nt −550, −221, and +4, respectively. A is wild-type codon 54 and B is the codon 54 variant.

Expression groups include haplotype pairs associated with high- or medium- and low- MBL expression. High-MBL expression haplotype pairs are HYA/HYA, HYA/LYA, HYA/LXA, LYA/LYA, and LYA/LXA; medium-MBL expression haplotype pairs are LXA/LXA, HYA/LYB, and LYA/LYB; and low-MBL expression haplotype pairs are LXA/LYB and LYB/LYB.

The *P* values and ORs (95% CI) are for the comparison between the homozygous wild-type genotype and the homozygous and heterozygous variant genotypes, for each polymorphism, and for the comparison of the haplotype pairs associated with high-MBL expression with those associated with medium- and low- MBL expression.

^a^Number of LL genotype/HL genotype/HH genotype in H/L locus, XX genotype/YX genotype/YY genotype in Y/X locus, QQ genotype/PQ genotype/PP genotype in P/Q locus, BB genotype/AB genotype/AA genotype in A/B locus, and low-MBL expression haplotype pairs/medium- MBL expression haplotype pairs/high-MBL expression haplotype pairs.

^b^The *P* values, ORs, and 95% CI are calculated in logistic regression with age and gender treated as fixed effects.

^c^The *P* values, ORs, and 95% CI are calculated in logistic regression with age and gender treated as random effects.

**Table 3:** Haplotypes for the four SNPs in *MBL2* gene and their associations with TB risk in Chinese.

Haplotypes	Cases, 2n (%) n = 2,040	Controls, 2n (%) n = 2,040	Score^a^	*P* value^a^	OR (95% CI)^b^
HYPA	990 (48.5)	1046 (51.3)	−2.36	0.018	1
LYPB	405 (19.9)	286 (14.0)	4.69	4.2 × 10^−6^	1.54 (1.27–1.87)
LXPA	268 (13.1)	296 (14.5)	−1.33	0.18	0.89 (0.72–1.10)
LYQA	234 (11.5)	270 (13.2)	−1.71	0.087	0.88 (0.70–1.09)
LYPA	143 (7.0)	142 (7.0)	0.020	0.98	1.04 (0.80–1.35)

OR, odds ratio; CI, confidence interval. The haplotype is in the order of H/L, Y/X, P/Q and A/B.

^a^Haplotype-specific scores and *P* values for each haplotype versus all others and the *P* value for global haplotype association are evaluated using the haplo.score-function. The global *P* value is 3.5 × 10^−5^.

^b^The ORs (95% CI) are calculated for haplotypes LYPB, LXPA, LYQA and LYPA using the haplo.glm-function, with haplotype HYPA as control.
